# The potential of *Terminalia chebula* in alleviating mild cognitive impairment: a review

**DOI:** 10.3389/fphar.2024.1484040

**Published:** 2024-10-18

**Authors:** Huimin Gao, Hanyu Lu, Nengqiao Fang, Jinsong Su, Rui Li, Wenjun Wang, Yi Zhang

**Affiliations:** ^1^ College of Pharmacy and Meishan Hospital, Chengdu University of Traditional Chinese Medicine, Chengdu, China; ^2^ School of Ethmic Medicine, Chengdu University of Taditional Chinese Medicine, Chengdu, Sichuan, China; ^3^ Research Institute of Integrated TCM and Western Medicine, Chengdu University of Traditional Chinese Medicine, Chengdu, China

**Keywords:** *Terminalia chebula*, MCI, Tibetan medicine, safety evaluation, phytochemistry, quality control, traditional medicinal uses

## Abstract

*Terminalia chebula* Retz. (*T. Chebula*, ཨ་རུ་ར།) is highly utilized in ethnic medicine. Its medicinal value is gradually being recognized and shows great potential in the improvement of mild cognitive impairment (MCI) disorders. Tibetan medicine theory classifies this type of disease as one of the “Jie Xie Syndrome (བརྗེད་བྱེད།).” The role of *T. Chebula* in such diseases has been increasingly studied. This work aimed to elucidate the research progress of *T. Chebula* in alleviating MCI. The review offers a critical update on the current understanding of the effect of *T. Chebula* on MCI and highlights new opportunities for exploring its therapeutic potential. This review discusses the role of *T. Chebula* in alleviating MCI and provides a comprehensive overview of the traditional medicinal uses, chemical composition, toxicology, and quality control aspects of *T. Chebula*. This review covers **171** chemical constituents and **11** active constituents targeting MCI, such as flavonoids, which can alleviate MCI, primarily through its antioxidative, anti-inflammatory, and neuroprotective properties. *T. Chebula* shows potential as a natural medicine for the treatment and prevention of MCI. As an important part of ethnomedicinal resources, this work offers valuable insights for future research on *T. Chebula*-containing ethnomedicines. Research on traditional drug treatments, optimized treatment standards, improved societal knowledge about MCI, and development of an early detection system is essential to the diagnosis and treatment of MCI. These efforts will provide better treatment resources for patients with MCI.

## Graphical Abstract

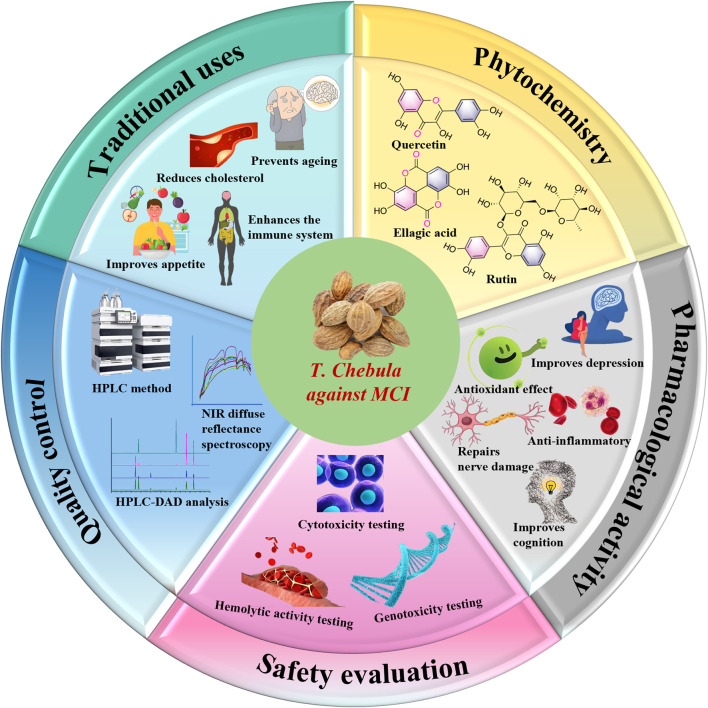

## Highlights

• *Terminalia chebula* has antioxidant, anti-inflammatory, repairing synaptic plasticity damage and neuroprotective properties for the treatment and prevention of mild cognitive impairment (MCI).
• *Terminalia chebula* is able to improve MCI.
• This review will be helpful for the succession and development of Tibetan medicine.

## 1 Introduction

Population aging is a significant aspect of social development and reflects the progress of human civilization. It has become a foundational national condition in China (Zhabokritsky et al., 2023). With the growing elderly population, cognitive disorders, such as dementia, have become a leading cause of death among seniors and present considerable health, medical, and socio-economic challenges (Bode et al., 2023; Ellen et al., 2017). Mild cognitive impairment (MCI), an intermediate stage between normal aging and dementia, involves a progressive decline in memory and cognitive function without meeting dementia criteria and poses a high risk for the progression of Alzheimer’s disease if left untreated (Petersen, 2011). Moreover, residing in high-altitude, low-oxygen environments, such as Tibet, can induce cognitive dysfunction and structural brain changes, especially above 4,000 m (Liu et al., 2023).

Tibetan medicine, an ancient tradition, has evolved into a distinct theoretical system over millennia (Dakpa, 2014). Understanding MCI through ethnomedical theories could complement modern clinical treatments. In Tibetan medicine, MCI is classified as “amnesia,” categorized under “Jie Xie Syndrome,” which is described in classic texts, such as Four Medical Tantras and the Blue Beryl and attributed to disturbances in “Long,” including a weak heart, excessive worry, and anxiety. Tibetan medicine addresses age-related diseases by preventing aging and tonifying Yang to regulate “Long” disorders and restore the body’s balance (Zhuoma and Renqing, 2020). The complexity of the etiology of MCI often complicates Western medical treatments, making them variable in efficacy and prone to adverse effects. Incorporating ethnomedical theories could aid in identifying effective ethnomedicines and potentially enhance MCI drug development (Yangmao, 2017; Campbell et al., 2018; Kessler et al., 2014; Lapeyre-Mestre, 2016; Matsunaga et al., 2019; Wang et al., 2022; Zhao et al., 2022).

*Terminalia chebula* Retz. (*T. Chebula*, ཨ་རུ་ར།), a prominent Tibetan medicinal herb, is renowned for its diverse therapeutic properties, including dispelling wind (qi), promoting blood circulation, and relieving toxicity. It is used for various conditions, from wind–heat rash to chronic diarrhea and epilepsy (Liu et al., 2018; Yang et al., 2017). Modern pharmacological studies have highlighted its antibacterial, antioxidant, hypoglycemic, antiviral, anti-inflammatory, and antitumor properties, thereby supporting its therapeutic potential, particularly in antioxidant and antidiabetic activities (Zhang et al., 2016).

While extensive previous research has detailed the pharmacological activities of *Terminalia chebula* (*T. Chebula*), there are not enough studies on its role in alleviating mild cognitive impairment. This review systematically elucidates the role of *T. Chebula* in mitigating MCI and provides a comprehensive overview of its traditional medicinal uses, chemical composition, toxicology, and quality control aspects. We emphasize pharmacological mechanisms and future research trends regarding *T. Chebula*’s potential as a natural medicine for the treatment and prevention of MCI. A thorough literature search was conducted across multiple databases, including CNKI, PubMed, and Web of Science, from January 1990 to March 2024, focusing on the chemical components and pharmacological activities of *T. Chebula*. The review highlights *T. Chebula*’s promise in alleviating MCI symptoms, offering essential insights and directions for the future development and application of *T. Chebula* in MCI treatment.

## 2 Traditional uses

**FIGURE 1:**
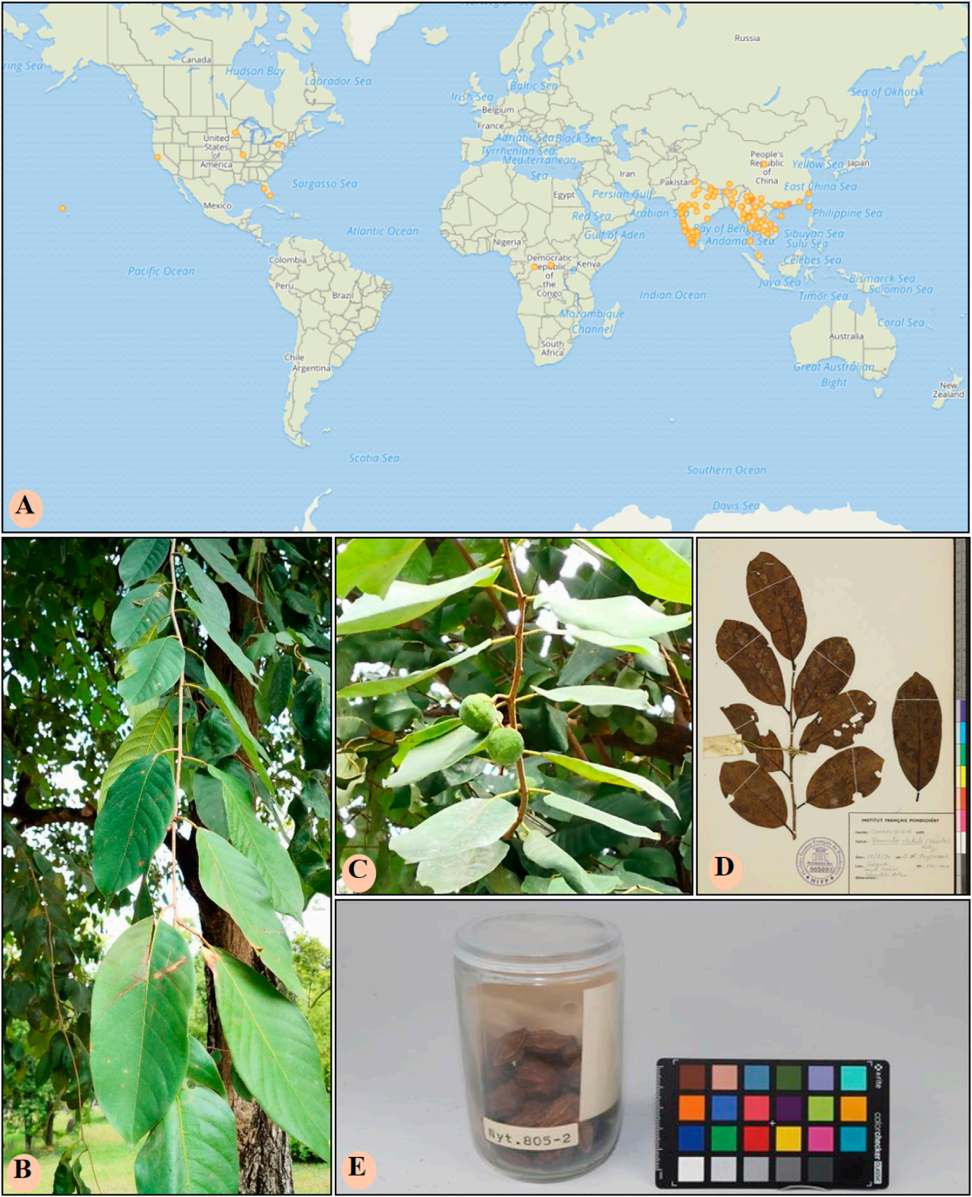
The geographical distribution and botanical morphology of *T. chebula*. **(A)** The geographical distribution of *T. chebula* in the world. **(B)** The leaf of *T. chebula.* **(C)** The fruit of *T. chebula.* **(D)** Specimen diagram of *T. chebula* leaves. **(E)** Specimen diagram of *T. chebula* fruit. The above pictures were all accessed through GBIF.org (https://www.gbif.org/) on 2023-05-22.

Traditional Tibetan medicine, *T. chebula*, refers to the dried fruit of a plant in the Combretaceae family. It is primarily grown in Malaysia, India, and Myanmar and is also found in various regions of China, including Yunnan, Tibet, Guangdong, and Guangxi (Ekambaram et al., 2020) (Figure 1). It has a few common names such as dark myrobalan, ink tree, or chebulic myrobalan (English), haritaki (Sanskrit and Bengali), Harad (Hindi), Harada (Marathi and Gujrati), Karkchettu (Telgu), and Kadukkaya (Tamil). It is notable as “haritaki” in Tibet. The synonyms of *T. Chebula* inciuding *T. parviflora* Thwaites, *T. reticulate* Roth, *T. tomentella* Kurz, *T. aruta* Buch. -Ham., ex G. Don, *T. zeylannica* Van Heurck and Muell. Arg (Bulbul et al., 2022). In Tibetan medicine, *T. Chebula* is typically combined with other herbs to treat various diseases. *T. Chebula* is extensively cited in ethnomedicine texts and is described in “Du Mu Ben Cao” as having thick leaves, with the fruit differing in traits and morphology; as such, it is classified into eight, seven, or five types. In Tibet, the dried fruit of *T. Chebula* is categorized into five kinds due to distinctive characteristics and is commonly used medicinally. Therapeutic and practical uses can vary depending on the processing method. Modern preparation methods include removing the seed, utilizing the pulp, and stir frying with either soil or bran (Rao and Nammi, 2006). *T. Chebula* is used to treat asthma, bronchitis, hepatitis, dyspepsia, eye diseases, hoarseness, and promote hair growth. The flesh of the plant has been used for the treatment of diarrhea, leprosy, and edema. It improves appetite, reduces cholesterol and blood pressure, strengthen the immune system, prevent aging, and enhance resistance against infections (Gupta et al., 2020). In clinical use, the therapeutic effects of specific preparations can be customized to treat various diseases and optimized by combining them according to distinct symptoms of cold and heat. Traditionally, *T. Chebula* is typically ingested as pills, powders, or decoctions (Table 1). In the *Four Medical Tantras*, *T. Chebula* is specifically described for its use in treating “Jie Xie Syndrome” (Feng et al., 2019).

**TABLE 1:** The prescription and Chinese patent medicine including *T. chebula*.

Prescription	Compositions	Clinical uses	Ref.
Hezi Wuwei Capsules	*T. chebula*, Punica granatum Linn., Semen Momordicae, Trogopterori Faeces, Hei-Bin-Pian	Strengthens the stomach and helps digestion, treats indigestion, liver and gallbladder fever, and jaundice	Pharmaceutical Standards of the Ministry of Health of the People’s Republic of China-Mongolian Medicines Branch Book, 1988. (《中华人民共和国卫生部药品标准·蒙药分册》1988年)
Sanzi granules	*T. chebula*, Gardenia jasminoides Ellis, Fructus Toosendan	It is used for distemper, dizziness, headache, blood heat, and redness of the eyes	Pharmaceutical Standards of the Ministry of Health of the People’s Republic of China-Mongolian Medicines Branch Book, 1988. (《中华人民共和国卫生部药品标准·蒙药分册》1988年)
Hezi Pi powder	*T. chebula*, Radix Aucklandiae, Coptis chinensis Franch., Glycyrrhizae Radix Et Rhizoma	Relieves abdominal pain	Baomin Ji (保命集)
Hezi drink	*T. chebula*, Semen Armeniacae Amarum., Tetrapanax papyriferus	Cures chronic cough without speech	Jisheng Fang (济生方)
Hezi pill	*T. chebula*, Os Draconis	Treating kidney deficiency and spermatorrhea	Puji Fang (普济方)
Appetizer powder	*T. chebula*, Panax ginsengC. A. Meyer, Glycyrrhizae Radix Et Rhizoma	Treating postpartum gastric deficiency and vomiting, chest fullness and inappetence	Chishui Xuanzhu (赤水玄珠)
Astringent intestinal powder	*T. chebula*, Halloysitum Rubrum, Os Draconis	Treatment of chronic dysentery in children	Baoying Zuoyao (保婴辑要)
Ersheng powder	*T. chebula*, Arecae Pericarpium	Treating paediatric wind-talking, shortness of breath and wheezing	Huoyou Xinshu (活幼心书)
Hezi soup	*T. chebula,* Platycodon grandifloras, Glycyrrhiza uralensis Fisch	Treatment of people who have lost their voice and are unable to speak	

## 3 Phytochemistry

*T. Chebula* contains a rich and diverse array of chemical constituents, which primarily include phenolic acids, tannins, triterpenoids, flavonoids, and volatile oils. A total of 171 compounds have been isolated from *T. Chebula* and comprise 83 tannins, 16 phenolic acids, 6 flavonoids, 29 triterpenoids, 17 volatile compounds, and 20 other compounds. This review focuses on the chemical constituents of *T. Chebula* and their potential role in improving the symptoms of MCI (Table 2).

**TABLE 2:** Chemical compounds of *T. chebula.*

Chemical class	Compounds	Structures	Ref.
Tannins	1,2,3-tri-*O*-galloyl-6-*O*-cinnamoyl-*β*-_D_-glucose	1	Lee et al. (2017)
	1,2,3,6-tetra-O-galloyl-4-O-cinnamoyl-*β*-_D_ -glucose	2	Lee et al. (2017)
	1,6-di-O-galloyl-2-O-cinnamoyl-*β*-_D_-glucose	3	Lee et al. (2017)
	1,2-di-O-galloyl-6-O-cinnamoyl-*β*-_D_-glucose	4	Lee et al. (2017)
	4-O-(2″,4″-di-O-galloyl-*α*-_L_-rhamnosyl) ellagic acid	5	Lee et al. (2017)
	Eschweilenol C	6	Lee et al. (2017)
	4-O-(4″-O-galloyl-*α*-_L_-rhamnosyl) ellagic acid	7	Lee et al. (2017)
	4-O-(3″,4″-di-O-galloyl-*α*-_L_-rhamnosyl) ellagic acid	8	Lee et al. (2017)
	Phyllanemblinin E	9	Lee et al. (2017)
	1′-O-methyl neochebulinate	10	Lee et al. (2017)
	Chebulanin	11	Juang et al. (2004)
	neochebulinic acid	12	Juang et al. (2004)
	Chebulic acid	13	Juang et al. (2004)
	6′-*O*-methyl chebulate	14	Lee et al. (2017)
	7′-*O*-methyl chebulate	15	Lee et al. (2017)
	Gallic acid	16	An et al. (2022)
	Methyl gallate	17	Lee et al. (2017)
	4-*O*-galloyl- (−)-shikimic acid	18	Lee et al. (2017)
	5-*O*-galloyl- (−)-shikimic acid	19	Ajala et al. (2014)
	1,3-di-*O*-galloyl-*β*-_D_-glucose	20	Lee et al. (2017)
	1,6-di-O-galloyl-*β*-_D_-glucose	21	Juang et al. (2004)
	1,3,6-tri-*O*-galloyl-*β*-_D_-glucose	22	Lee et al. (2017)
	1,2,3,6-tetra-O-galloyl-*β*-D-glucose	23	Lee et al. (2017)
	1,3,4,6-tetra-O-galloyl-*β*-_D_-glucose	24	Lee et al. (2017)
	1,2,3,4,6-penta-O-galloyl-*β*-_D_-glucose	25	Juang et al. (2004)
	6-0-galloyl-_D_-glucose	26	Lee et al. (2017)
	3,6-di-*O*-digalloyl-_D_-glucose	27	Lee et al. (2017)
	3,4,6-tri-*O*-galloyl-_D_-glucose	28	Lee et al. (2017)
	Corilagin	29	Kim et al. (2022)
	Tercatain	30	Lee et al. (2017)
	Gemin D	31	Lee et al. (2017)
	Tellimagrandin I	32	Lee et al. (2017)
	Digallic acid	33	Lee et al. (2017)
	Punicacortein C	34	Lee et al. (2017)
	Punicacortein D	35	Lee et al. (2017)
	Terflavin A	36	Singh et al. (2016)
	Phyllanemblinin F	37	Lee et al. (2017)
	Brevifolin carboxylic acid	38	Lee et al. (2017)
	Punicalagin	39	Pfundstein et al. (2010)
	1′-O-methyl neochebulanin	40	Kim et al. (2018)
	2-O-cinnamoyl-1,6-di-O-galloyl-*β*-_D_-glucose	41	Kim et al. (2018)
	1,3,4,6-tetra-O-galloyl-2-O-cinnamoyl-*β*-_D_-glucose	42	Kim et al. (2018)
	Dimethyl 4′-epi-neochebulagate	43	Kim et al. (2018)
	1-O-galloyl-6-O-cinnamoyl glucose	44	Wang (2018)
	Chebuloside Ⅱ	45	Wang (2018)
	1,4-di-O-galloyl-*β*-_D_-glcose	46	Wang (2018)
	Tellimagrandin	47	Wang (2018)
	Pentagalloyl glucose	48	Wang (2018)
	(−)-shikmide 4-O-gallate	49	Wang (2018)
	(−)-shikimide 3-O-gallate	50	Wang (2018)
	(−)-shikmide 5-O-gallate	51	Wang (2018)
	2,3-(S)-HHDP -_D_-glucose	52	Wang (2018)
	1,2,6-tri-O-galloyl-*β*-_D_-glucose	53	Wang (2018)
	1-O-galloyl-2-4-chebuloyl-*β*-_D_-glucopyranose	54	Liu et al. (1998)
	Hamamelitannin	55	Li et al. (2019)
	Dimethyl neochebulinate	56	Kim et al. (2018)
	Phyllanemblinin F	57	Kim et al. (2018)
	Eugeniin	58	Li et al. (2019)
	Ellagic acid	59	Zhang et al. (2018)
	Terchebin	60	Zhang et al. (2018)
	Chebulinic acid	61	Zhang et al. (2018)
	Neochebulagic acid	62	Kim et al. (2018)
	6′-*O*-Methyl-neochebulagate	63	Kim et al. (2018)
	Dimethyl-neochebulagate	64	Kim et al. (2018)
	Methyl chebulagate	65	Kim et al. (2018)
	3,6-di-O-galloyl-_D_-glucose	66	Ding et al. (2001)
	6-O-galloyl-_D_-glucose	67	Ding et al. (2001)
	Terflavin B	68	Pfundstein et al. (2010)
	Chebulagic acid	69	Manosroi et al. (2010)
	Terchebulin	70	Lin et al. (1990)
	Glucogallin	71	Yang (2016)
	Casuarinin	72	Zhou (2020)
	Chebumeinin B	73	Wang (2018)
	Euphormisin M3	74	Li et al. (2019)
	Punicalagin A	75	Zhang et al. (2001)
	Punicalin	76	Lin et al. (1990)
	Punicalagin B	77	Zhang et al. (2001)
	HHDP-glucose	78	Zhang et al. (2001)
	Digalloylglucose	79	Zhang et al. (2001)
	Phyllanemblinin D/Isomer	80	Zhang et al. (2001)
	Tetragalloylglucose	81	Zhang et al. (2001)
	Pentagalloylglucose	82	Zhang et al. (2001)
	Isoterchebulin	83	Manosroi et al. (2010)
Phenolic acid	Ethyl gallate	84	Zhang et al. (2001)
	Shikimic acid	85	Zhou et al. (2020)
	Triethylchebulaie	86	Lu et al. (1991)
	Methyl Shikimate	87	Zhang et al. (2001)
	*trans*-Cinnamic acid	88	Zhang et al. (2001)
	Protocatechuic acid	89	Zhang et al. (2001)
	Caftaric acid	90	Li et al. (2019)
	2,4-Dihydroxybenzoic acid	91	Li et al. (2019)
	m-galloylgallic acid	92	Li et al. (2019)
	Chebulin	93	Sornwatana et al. (2015)
	Digallice acid	94	Kim et al. (2018)
	11-Methyl-chebulate	95	Kim et al. (2018)
	13-Methyl-chebulate	96	Kim et al. (2018)
	11,12-Dimethylchebulate	97	Wang (2018)
	Brervifolincaboxylic acid	98	Li et al. (2022)
	Quinic acid	99	Pfundstein et al. (2010)
Triterpenoid	Terminoic acid	100	Lu et al. (1992)
	Arjugenin	101	Singh et al. (2016)
	Arjunolic acid	102	Lu et al. (1992)
	Chebupentol	103	Lu et al. (1992)
	Quercotriterpenoside I	104	Kim et al. (2018)
	Terminolic acid	105	Kim et al. (2018)
	23-galloyl-arjunolic acid	106	Conrad et al. (1998)
	Arjunetin (24-deoxy-sericoside)	107	Kim et al. (2018)
	23-galloylarjunolic-acid-28-O-*β*-_D_-glucopyranosyl ester	108	Kim et al. (2018)
	Arjunic acid	109	Kim et al. (2018)
	Pinfaenoic-acid-28-O-*β*-_D_-glucopyranosyl ester	110	Kim et al. (2018)
	Chebuloside-Ⅰ	111	Li et al. (2019)
	Arjunglucoside	112	Zhang et al. (2001)
	β-sitosterol	113	Lu et al. (1992)
	Chebuloside-Ⅱ	114	Zhang et al. (2018)
	2α-hydroxymicromeric acid	115	Zhang et al. (2018)
	Maslinic acid	116	Asif et al. (2019)
	2α-hydroxyursolic acid	117	Zhang et al. (2018)
	Daucosterol	118	Yang et al. (2008)
	Ajunglucoside IV	119	Wang et al. (2010)
	Ajunglucoside V	120	Wang et al. (2010)
	Quadranoside I	121	Wang et al. (2010)
	Arjunglucoside I	122	Wang et al. (2010)
	Arjunglucoside II	123	Wang et al. (2010)
	Arjunglucoside III	124	Wang et al. (2010)
	Arjunetin	125	Wang et al. (2010)
	Sericoside	126	Wang et al. (2010)
	Bellericoside	127	Wang et al. (2010)
	2α,19α-Dihydroxy-3-O-12-en-28-ursolic-acid-O-*α*-_L_-rhamnose-O-*β*-_D_-glucoside	128	Liu et al. (2012)
Flavonoid	Quercetin	129	Yang and Tang (2012)
	Quercetin-3-O-rhamnoside	130	Yang and Tang (2012)
	Rutin	131	Yang and Tang (2012)
	Quercetin-dihydrate	132	Yang and Tang (2012)
	Kaempferol-3-O-rutinoside	133	Zhang et al. (2001)
	5,7,2′-Tri-O-methylflavone-4′-O-*β*-_D_-galactosyl-O-*β*-_D_-glucoside	134	Liu et al. (2012)
Volatile	Tetradecanoic acid	135	Jokar et al. (2016)
	Palmitic acid	136	Jokar et al. (2016)
	Linoleic acid	137	Jokar et al. (2016)
	Oleic acid	138	Jokar et al. (2016)
	Octadecadienoic acid	139	Jokar et al. (2016)
	Benzoic acid	140	Lian et al. (2008)
	Pentadecane	141	Lian et al. (2008)
	Hexadecane	142	Lian et al. (2008)
	Heptadecane	143	Lian et al. (2008)
	Octadecane	144	Lian et al. (2008)
	Eicosane	145	Lian et al. (2008)
	9,12-Octadeeadienoic acid (z, z)	146	Lian et al. (2008)
	Myristic acid	147	Lian et al. (2008)
	2, 6-Bis (1, 1-dimthylethyl) -4-methyl-Phenol	148	Lian et al. (2008)
	*Cis*-α-Santalol	149	Lian et al. (2008)
	2, 6-Dimethyl Heptadecane	150	Lian et al. (2008)
	Hexadecanoic acid	151	Lian et al. (2008)
Others	L-aspartic acid	152	Lin et al. (1996)
	L-glutamic acid	153	Nigam et al. (2020)
	L-arginine	154	Lin et al. (1996)
	L-lysine	155	Barthakur and Arnold (1991)
	Proline	156	Nigam et al. (2020)
	Fructose	157	Nigam et al. (2020)
	Glucose	158	Lin et al. (1996)
	Sucrose	159	Nigam et al. (2020)
	β-arabinose	160	Lin et al. (1996)
	Rhamnose	161	Barthakur and Arnold (1991)
	Mannitol	162	Lin et al. (1996)
	Tricarboxylic acid	163	Nigam et al. (2020)
	Soft-fatty-acids	164	Barthakur and Arnold (1991)
	Carotene	165	Lin et al. (1996)
	*trans*-Cinnamic acid	166	Yang et al. (2008)
	Hexadecane-d34	167	Lin et al. (1996)
	Butylated hydroxytoluene	168	Lin et al. (1996)
	3-O-methylellagic acid 4′-O-*α*-_L_-rhamnopyranoside	169	Wang et al. (2010)
	Sennoside A	170	Cai et al. (2008)
	Arachidic acids	171	Cai et al. (2008)

### 3.1 Tannin-based

**FIGURE 2:**
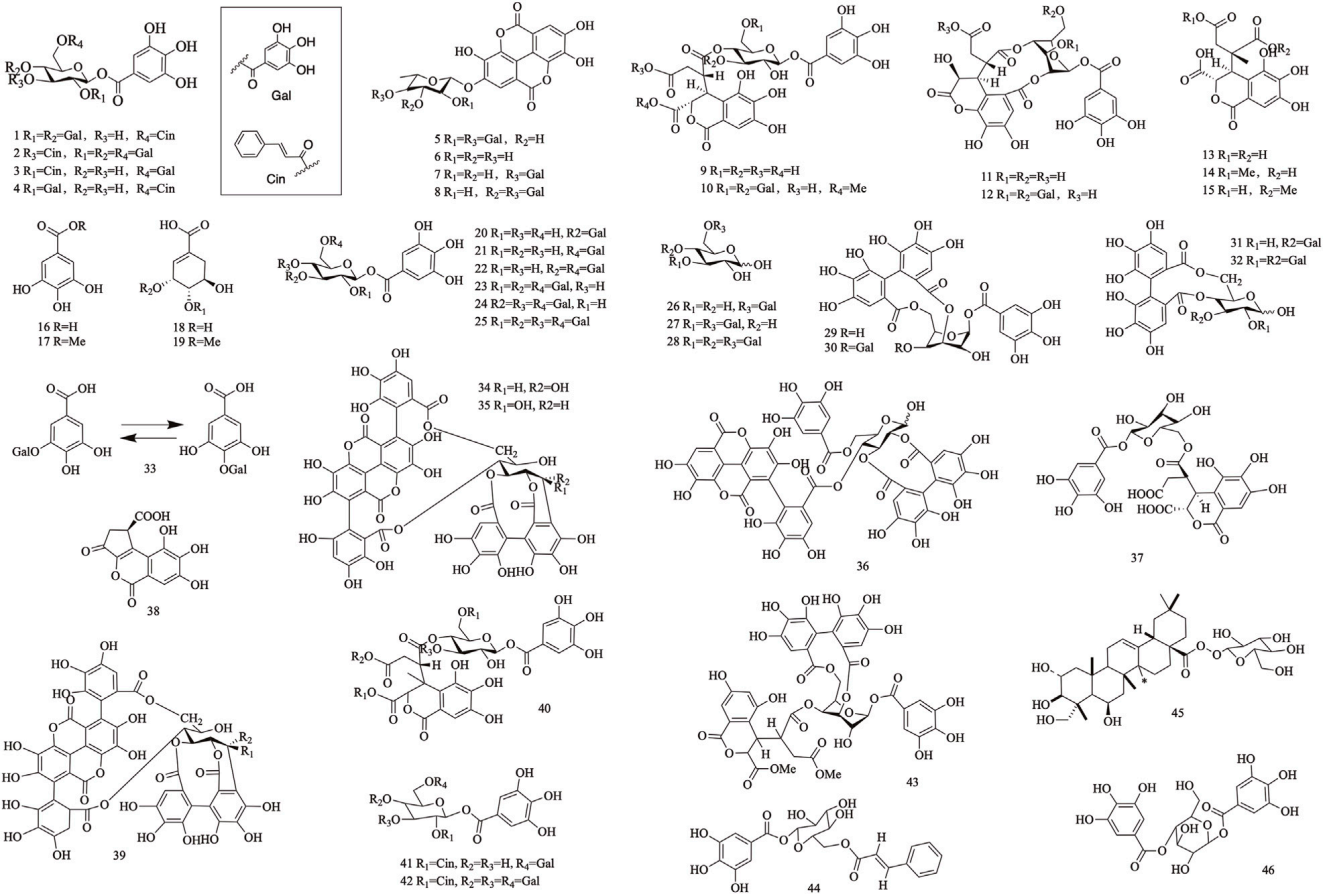
The structures of Tannin (compound **1**–**46**).

**FIGURE 3:**
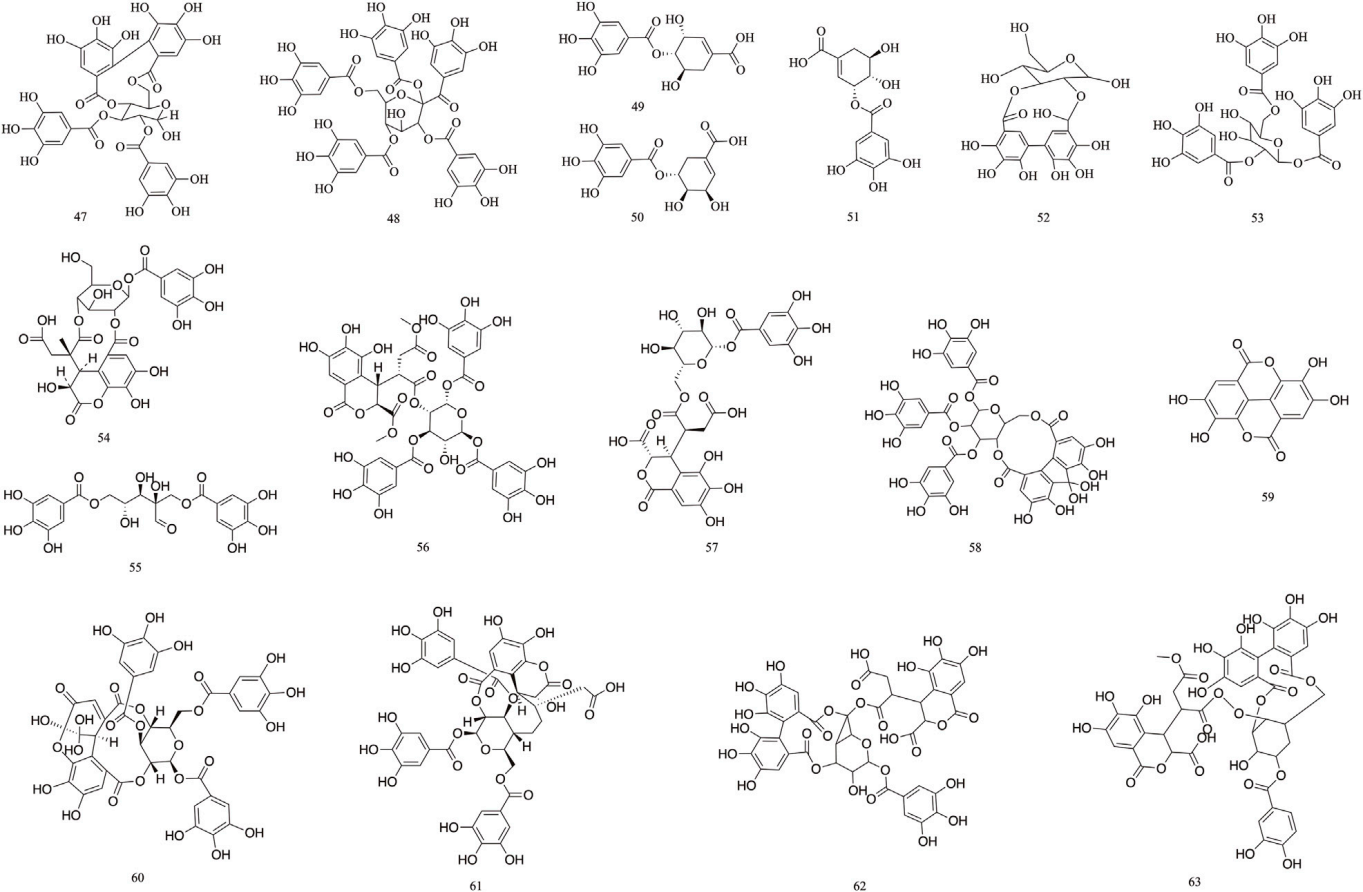
The structures of Tannin (compound **47**–**63**).

**FIGURE 4:**
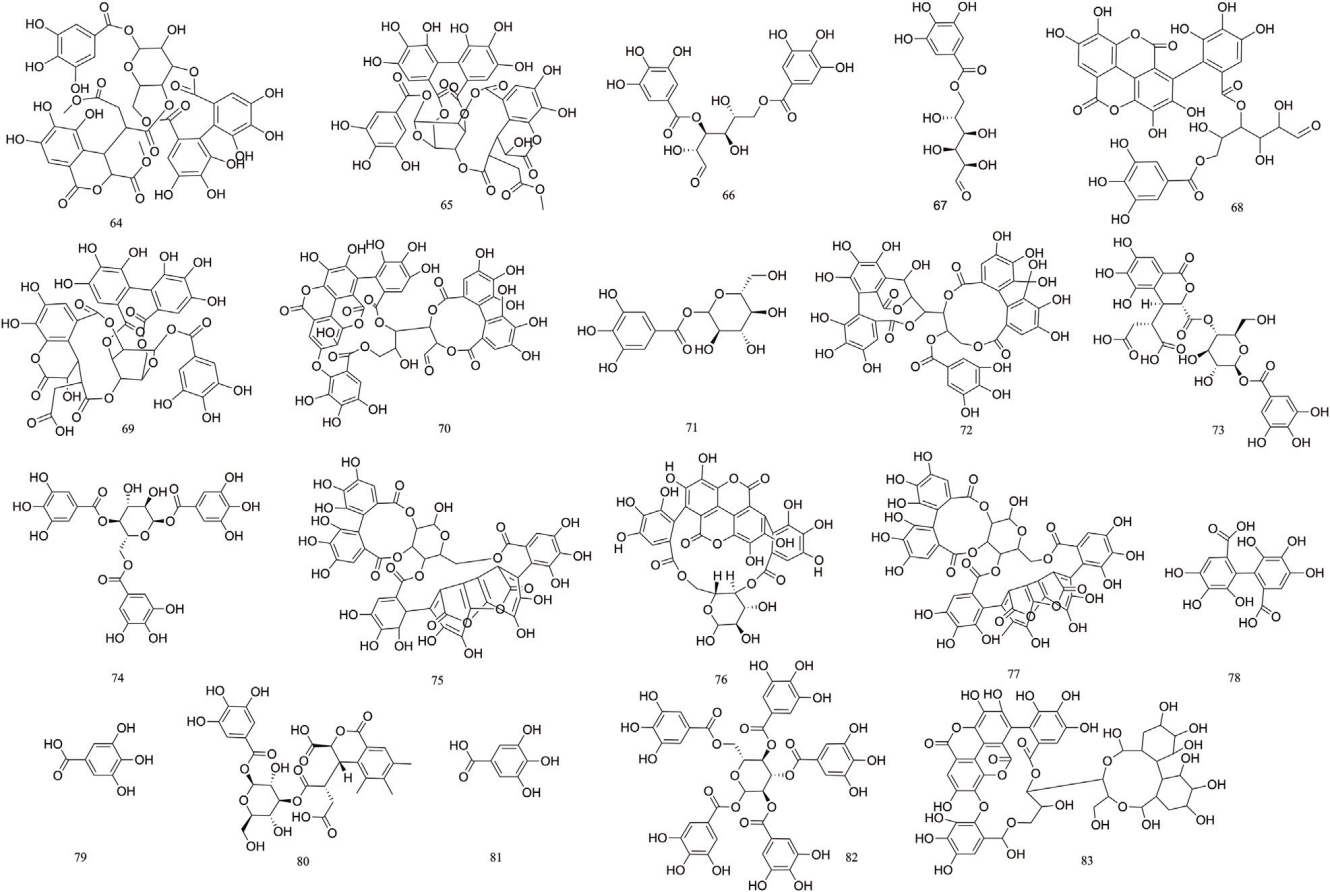
The structures of Tannin (compound **64**–**83**).

Tannins are polyphenolic compounds classified into hydrolyzable and condensed types, with gallic acid and glucose as their structural units. *T. Chebula* contains a substantial amount of tannin compounds, comprising 23.6%–37.36% of its composition. A total of 83 tannin compounds have been extracted and isolated from *T. Chebula* (Reddy et al., 1994; Li et al., 2019). Seven polyphenolic compounds were isolated from *T. Chebula* (Ding et al., 2001). Ellagic acid **59**, terchebin **60**, chebulinic acid **61**, corilagin **29**, and punicalagin **39** were isolated from *T. Chebula* (Lin et al., 1990). Chebulagic acid **69** and terchebulin **70** were identified from the ethyl acetate extract of *T. Chebula* (Lee et al., 2007). The components currently isolated from *T. chebula* are glucogallin **71**, casuarinin **72**, chebulanin **11**, chebumeinin B **73**, punicalagin A **75,** and punicalagin B **77** (Jeong et al., 2019; Yao et al., 2023; Zhao et al., 2022). Some of these key active ingredients play an important role in improving MCI (Figures 2–4).

### 3.2 Phenolic acid

**FIGURE 5:**
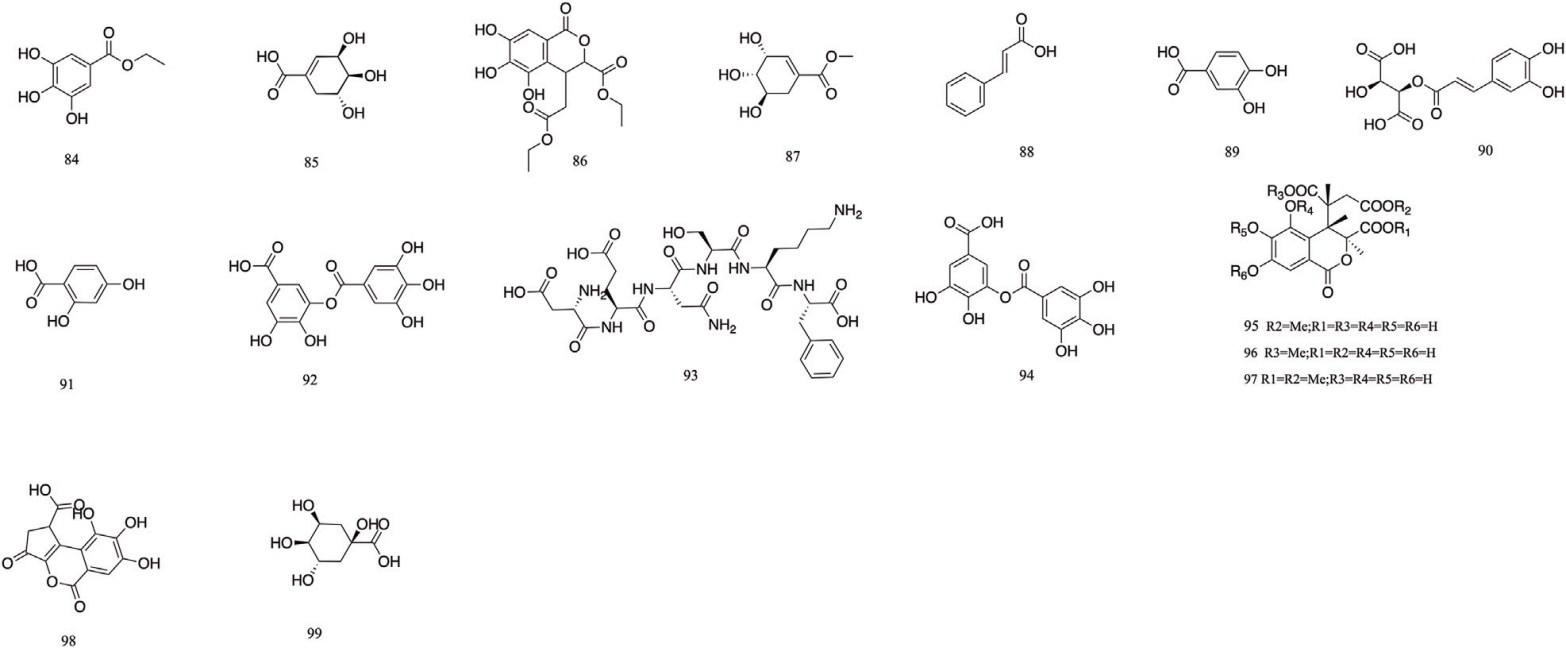
The structures of Phenolic acid (compound **84**–**99**).

*T. Chebula* contains a large amount of phenolic acids. These compounds contain a large number of active sites in their structure and have a variety of biopharmacological activities, such as anti-inflammatory, antiviral and immunomodulatory effects, *etc.* Phenolic compounds are also excellent antioxidants and their phenolic hydroxyl groups have obvious scavenging effects on free radicals such as peroxyl radicals (OOH) and hydroxyl radicals (OH), which can cause structural and functional damage to the membranes of biological tissues due to peroxidation (Chang and Lin, 2012). *T. Chebula* contains trihydroxybenzoic acid and dihydroxybenzoic acid (Walia et al., 2011). A total of 16 phenolic acids have been compiled (**84–99**). Ethyl gallate **84**, shikimic acid **85**, methyl shikimate **87**, trans-cinnamic acid **88**, and 2,4-Dihydroxybenzoic acid **91** were isolated from *T. Chebula* (Zhi et al., 2020). Quinic acid **99** was also identified. Furthermore, 11-methyl chebulate **95**, 13-methyl chebulate **96**, 11,12-dimethylchebulate **97**, and brervifolincaboxylic acid **98** were isolated from *T. Chebula* (Pfundstein et al., 2010). Other compounds were also isolated from *T. Chebula* (Lu et al., 1991) (Figure 5).

### 3.3 Triterpenoid

**FIGURE 6:**
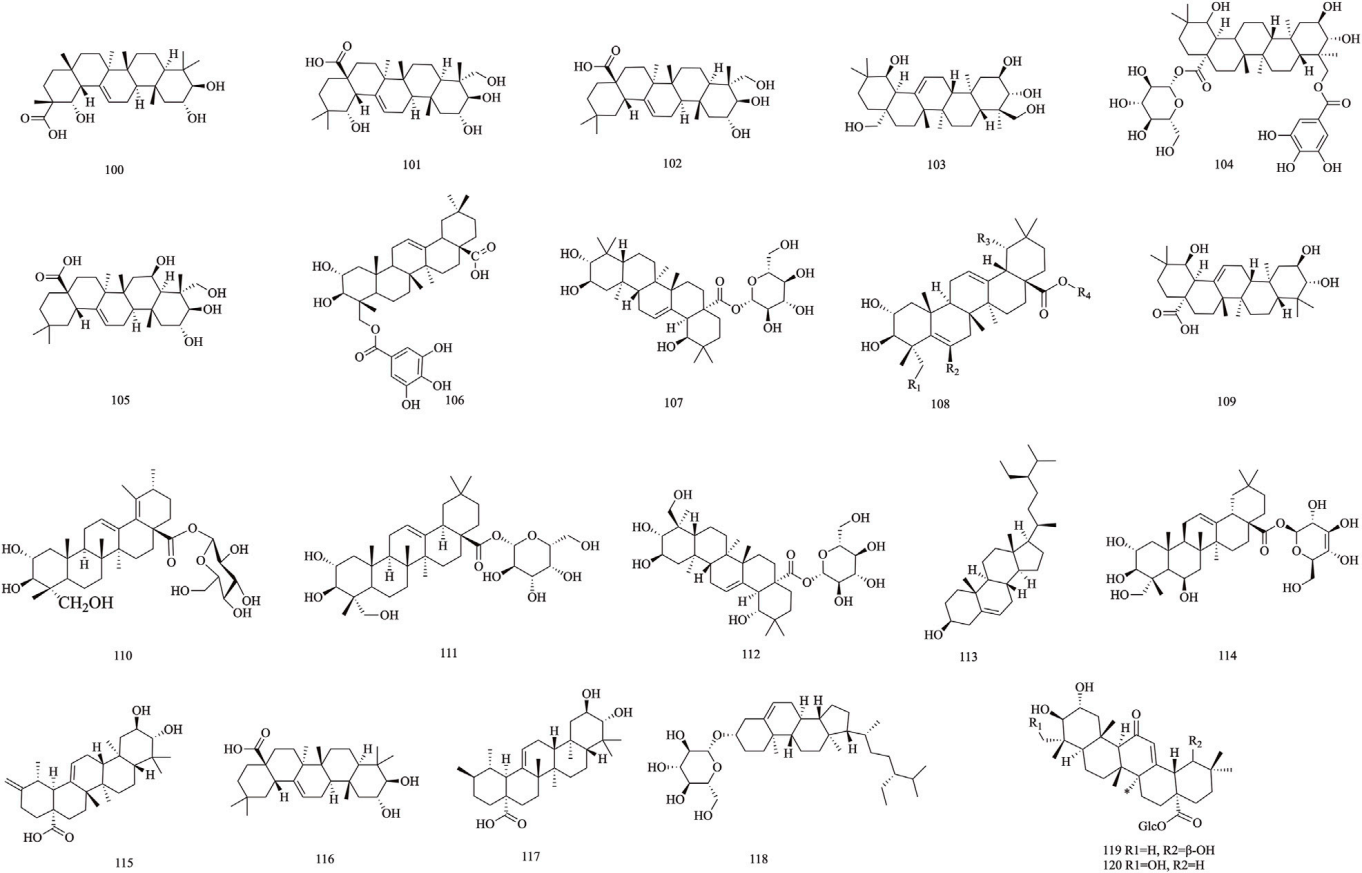
The structures of Triterpenoid (compound **100**–**120**).

**FIGURE 7:**
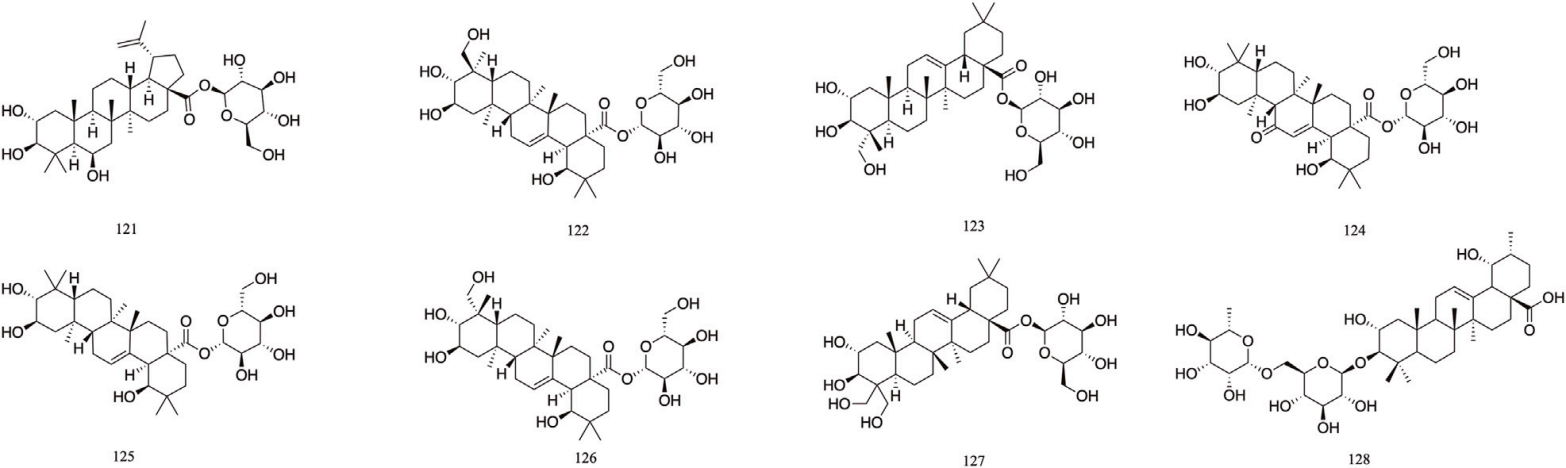
The structures of Triterpenoid (compound **121**–**128**).

*T. Chebula* mainly contains pentacyclic triterpenes and their glycoside components. Triterpene glycosides typically feature hydroxyl groups at C-2 and C-3, angular methyl groups at C-8, C-10, and C-14, and double bonds at C-12 and C-13. Triterpenoids exhibit a range of biological activities, including hypoglycemic, anti-tumor, antioxidant, hepatoprotective, antibacterial, renal, and immune system-regulating effects. These compounds demonstrate neuroprotective properties through various molecular mechanisms, encompassing the modulation of neuroinflammation, oxidative stress, and autophagy. Furthermore, they may mitigate neurological disorders by improving mitochondrial function and inhibiting endoplasmic reticulum stress (Liu et al., 2012). A total of 29 triterpenoids (**100**–**128**) have been identified. Triterpenoids isolated from *T. Chebula* fruits include arjunic acid **109**, arjugenin **112**, chebupentol **103**, maslinic acid **116**, 2α-hydroxyursolic acid **115**, and daucosterol **118** (Manosroi et al., 2010) (Figures 6, 7).

### 3.4 Flavonoid

**FIGURE 8:**
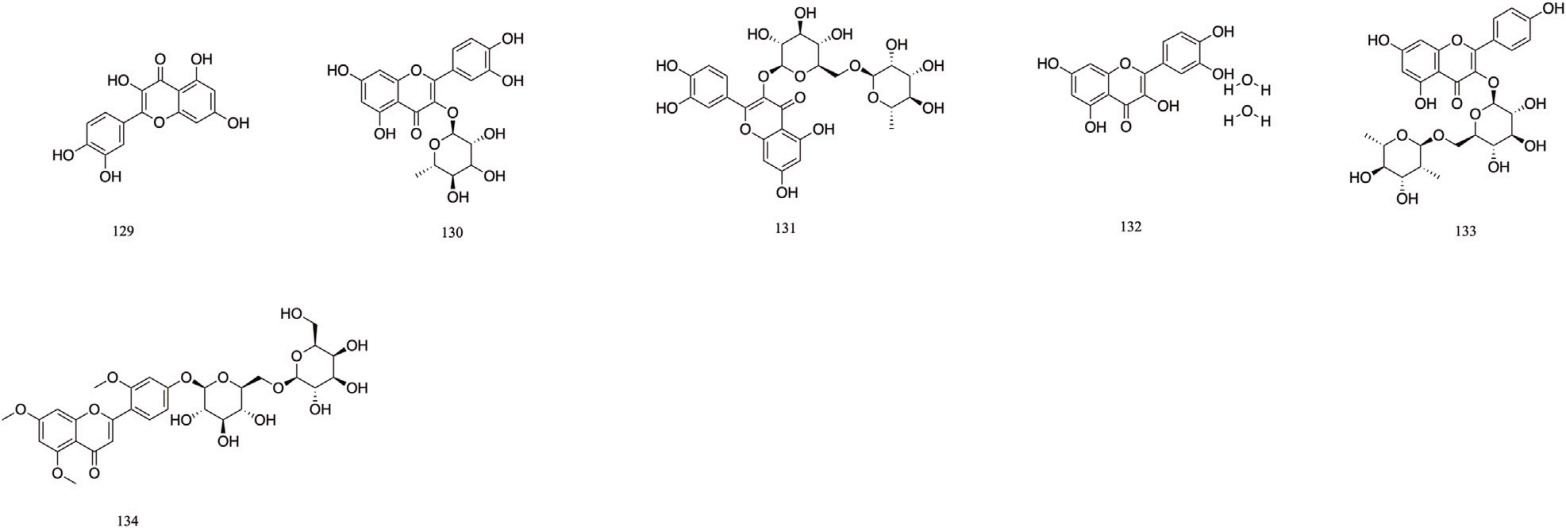
The structures of Flavonoid (compound **129**–**134**).

The majority of flavonoids are derivatives containing hydroxyl groups and often have methoxy or other substituents on the parent nucleus. Seven major flavonoid compounds (**129**–**134**) have been identified, including quercetin (**129**), quercetin-3-O-rhamnoside (**130**), and rutin (**131**). Components **130**, **131**, and **132** were isolated from the fruits of *T. chebula* (Tang et al., 2012). Several *in vitro* studies have demonstrated the neuroprotective effects of compound **129** and its potential to enhance cognitive performance clinically (Khan et al., 2019) (Figure 8).

### 3.5 Volatile

**FIGURE 9:**
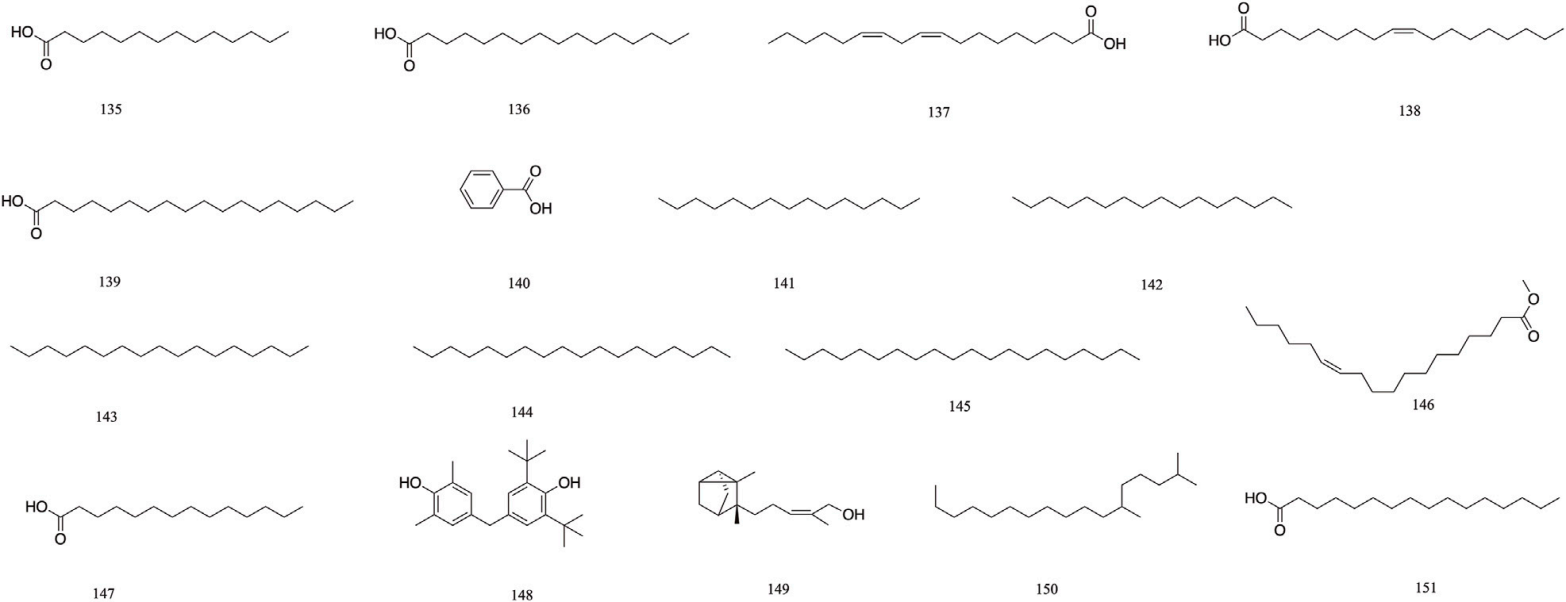
The structures of Volatile (compound **135**–**151**).

Volatile components primarily consist of fatty acids. *T. Chebula* also contains volatile components. A total of 16 volatile components have been identified (**135–151**), such as tetradecanoic acid **135**, palmitic acid **136**, linoleic acid **137**, oleic acid **138**, and octadecadienoic acid **139** (Jokar et al., 2016). Gas–mass spectrometry was employed to analyze the volatile components of *T. Chebula* (Lin et al., 1996) (Figure 9).

### 3.6 Other components

**FIGURE 10:**
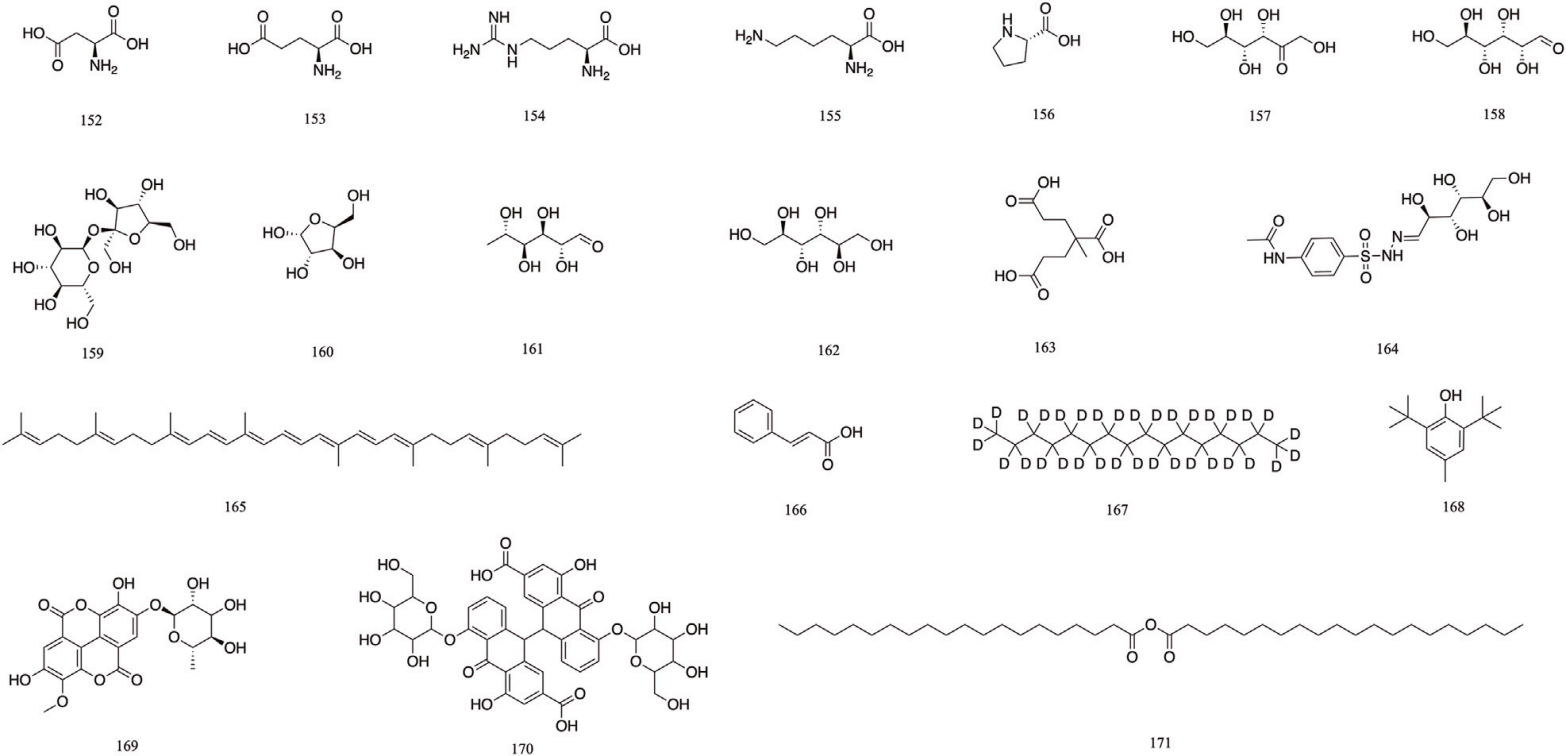
The structures of other components (compound **152**–**171**).

The 19 other compounds (**152–171**) found in *T. Chebula* include amino acid compounds such as L-aspartic acid **152**, L-glutamic acid **153**, L-arginine **154**, L-lysine **155**, and proline **156**; sugar compounds such as fructose **157**, glucose **158**, sucrose **159**, β-arabinose **160**, and rhamnose **161**; aliphatic compounds such as mannitol **162**, tricarboxylic acid **163**, soft fatty acids **164**, and carotene **165** (Rai and Joshi, 2009; Nigam et al., 2020) (Figure 10).

## 4 Pharmacological activity of *T. Chebula* to improve MCI

**FIGURE 11:**
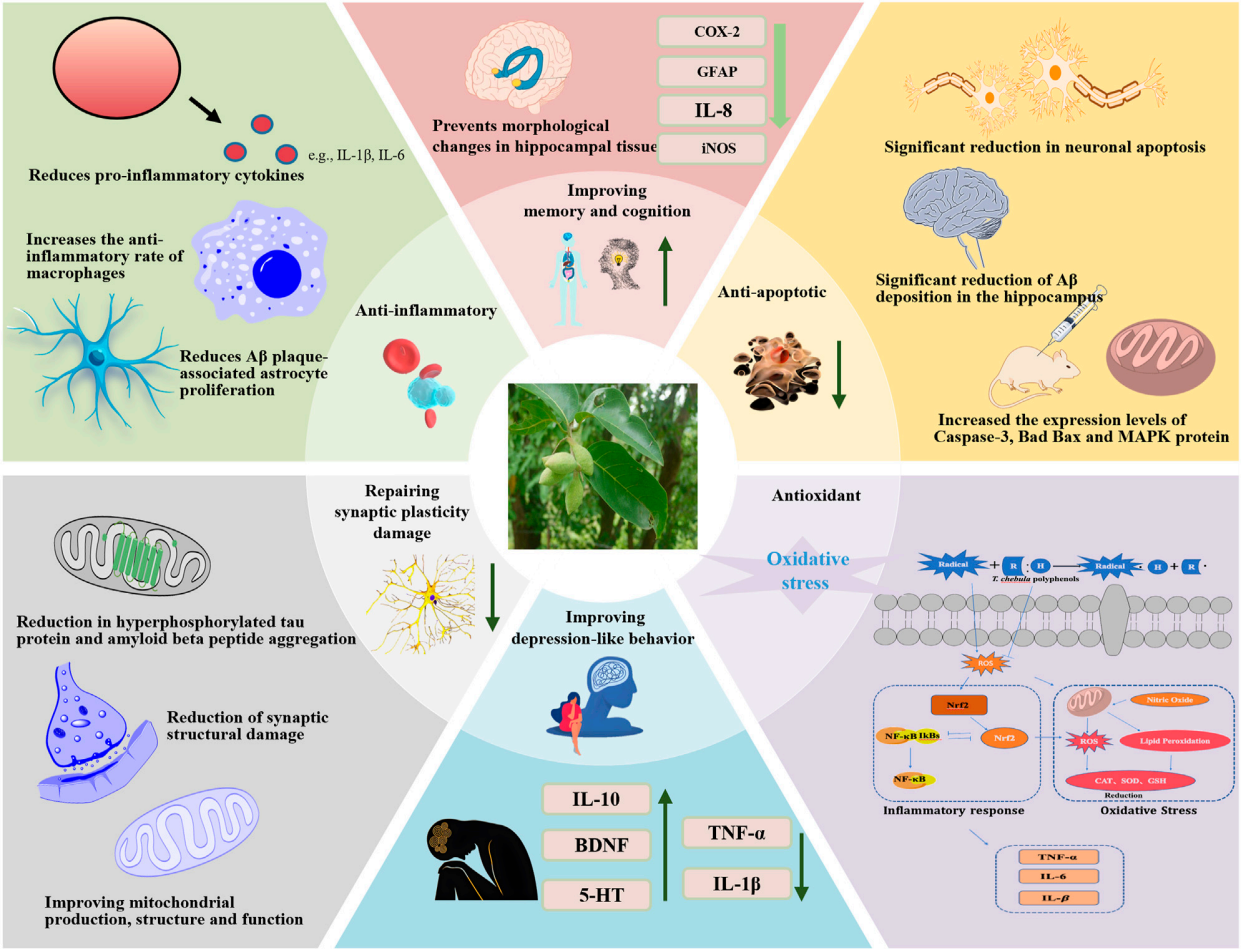
Molecular mechanism of MCI alleviation by *T. chebula*. Abbreviations: IL-6, interleukin-6; IL-1β, interleukin-1β; TNF-α, tumor necrosis factor-α; ROS, reactive oxygen species; Nrf2, nuclear factor erythroid 2-related factor 2; CAT, catalase; SOD, superoxide dismutase; GSH, Glutathione; NF-κB, nuclear transcription factor-κB; MAPK, mitogen-activated protein kinase.

Various components of *T. Chebula* possess multiple pharmacological activities, mainly including anti-inflammatory, antioxidant, neuroprotective, cognitive enhancement, antimicrobial, anti-tumor, and angiogenesis effects (Bulbul et al., 2022) (Figure 11). The pharmacological effects aimed at improving MCI symptoms are particularly notable (Tables 3, 4).

**TABLE 3:** Animal behavioral tests representing the effects of *T. chebula* against MCI.

Sl./No.	Animal model	Dosage and treatment duration	Behavioral tests	References
I	Improvement of depression-like behavior			
1	young (age of 8–12 weeks) male Swiss albino mice	*T.chebula* extract (100 or 200 mg/kg, p.o.) for 15 days	Forced swim test (FST), Tail suspension test (TST) and field test	Mani et al. (2021)
2	Wistar rats	*T.chebula* extract (18 mg/kg, p.o.)	Elevated Plus Maze test	Chandrashekar et al. (2013)
3	ICR mice	*T.chebula* extract (390, 780 and 1560 mg/kg, p.o.) for 7 days	FST and TST	Dattatray et al. (2014)
4	CUMS depression model mice	EA (100 and 20 mg/kg, p.o.) for 6 weeks	Sucrose preference (SPT), TST and FST	Huang (2020)
5	Balb/c mice	Tannin rich extract of *T. chebula* (25, 50 and 100 mg/kg, p.o.) for 21 days	Elevated maze (EPM), open Field test (OFT), light/camera obscurium Test (LDT) and Vogel’s Conflict Test (VCT)	Chandrasekhar et al. (2018)
II	Improvement of memory and cognitive level			
1	Wild-type adult (<8 months old) zebrash of both sexes	Quercetin and Rutin pretreatments (50 mg/kg, single injection, i.p.)	Inhibitory avoidance test	Richetti et al. (2011)
2	ICV-STZ rats	Rutin (25 mg/kg, orally) for 3 weeks	Morris Water Maze test	Javed et al. (2012)
3	ICV-STZ rats	Rutin (10, 25 and 50 mg/kg, i.g.) for 30 days	Passive avoidance learning and memory test	Hasanein et al. (2020)
4	Sprague-Dawley rats	Rutin hydrate (50 mg/kg in 0.5 mL saline, intraperitoneal injection) for 12 weeks	Morris water maze test; Delayed alternation task in the T-maze; Passive avoidance test	Qu et al. (2014)

**TABLE 4:** Molecular mechanism of MCI alleviation by *T. chebula* (*T. Chebula*).

Sl./No.	Model	Dosage and treatment duration	Molecular mechanism	References
A	Antioxidant effect			
1	Wistar rats	*T. Chebula* extract (100–500 μg m L^-1^, p.o.)	CAT, superoxide dismutase (SOD) ↑; GSH content in rats, lipid peroxide (LPO) production ↓	Saha and Verma (2015)
2	Neuroinflammation induced by LPS in Wistar rats	EA (100 mg/kg, i.p.) for 8 days	Glial cell expression ↑; Phosphorylated Tau and oxidative damage↓; Preventing the increase of acetylcholinesterase activity	Dornelles et al. (2020)
3	Sleep deprivation-induced memory impairment and anxiety C57BL/6J mice	EA (50和100 mg/kg, i.p.) for 21 days	SOD, GPx, dendritic spine density in the hippocampus, neuron survival ↑; MDA, IL-1β, IL-6, TNF-α ↓; Inhibiting TLR4 and activating Nrf2 pathway	Wang et al. (2020a)
B	Anti-inflammatory effect			
1	Sprague-Dawley (SD) rats	Quercetin and Rutin pretreatments (50 mg/kg, single injection, i.p.)	Relate to antioxidant activity, while the component absorbed in the brain is related to its neuroprotective effect	Zhang et al. (2017)
2	Falling weight strike induced traumatic brain injury in Wistar rats	GA (100 mg/kg, P.O.) for 7 days	IL-1β, IL-6, TNF- α, MDA ↓	Mirshekar et al. (2018)
3	LPS induces inflammation in RAW264.7 cell	Corilagin (20 ng/mL, 10 ng/mL and 2 ng/mL) for 24 h	HO-1 ↑; TNF-α, IL-1β, IL-6, NO (iNOS) ↓; Blocking NF-κB nuclear translocation	Zhao et al. (2008)
4	Reoxygenation induced rat pheochromocytoma (PC12) cells death; H_2_O_2_ induced oxidative stress in PC12 cells; LPS induced inflammation in microglia cells	*T. Chebula* extract (0.01, 0.1, 1 μg/mL)	PC12 cells viability, microglia cells viability ↑; MDA in PC12 cells, NO in microglia cells ↓	Gaire et al. (2013)
	LPS induced inflammation in microglia cells	*T. Chebula* extract (0–80 μg/mL)	Arg-1, urea ↑; TNF-α, IL-1β, IL-6, PGE-2, COX-2, NO ↓	Rahimi et al. (2018)
C	Repairing synaptic plasticity damage			
1	Hypobaric oxygen chamber induced memory impairment in SD rats	Quercetin (50, 75 and 100 mg/kg, bw/d) for 7 days	Learning and memory abilities of rats, mitochondrial generation in rat hippocampus ↑; Mitochondrial structural damage and euronal synaptic structure damagein rat hippocampus; Regulating SIRT1/PGC-1α/Nrf-1/Tfam and PGC-1α/FNDC5/BDNF pathway	Liu (2016)
2	Chronic lead exposure caused synaptic plasticity damage in the DG region of Wistar rats	Quercetin (30 mg/kg, bw/d)for 7 days	Synaptic transmission efficiency ↑; Lead content in blood and hippocampus tissue, synaptic plasticity damage, LTP damage in DG region ↓	Hu (2008)
3	Glutamate-induced PC12 cells damage	Quercetin (0.5,1,5 and 10 μmol/L) for 24 or 48 h	Transient outward potassium current (IA), rectified potassium current (IK) ↓	Zhao et al. (2010)
D	Anti-apoptotic effect			
1	Permanent middle cerebral artery occlusion in Sprague Dawley rats; H_2_O_2_ induced PC12 cells death	Quercetin (30 mg/kg, i.p.)	Expression of survival signals↑; brain ischemic lesion ↓	Dajas et al. (2003)
2	HCT-15, COLO-205, MDA-MB-231, DU-145 and K562 cell lines	*T. Chebula* extract (20 mg/mL)	COX-1, COX-2, 5-LOX, cell apoptosis ↓	Reddy et al. (2009)
3	APP/PS1 double transgenic mice	EA (50 mg/kg/day, i.g.) for 60 days	Learning and memory abilities of mice ↑; Neuronal apoptosis, Aβ deposition, hyperphosphorylation of tau ↓	Zhong et al. (2018)
4	Isoflurane-induced neuroapoptosis in SD rat pups	Rutin (10, 20 or 40 mg/kg body weight, b.wt, orally)for 15 days	Learning and memory abilities of rat pups ↑; neuroapoptosis ↓	Li et al. (2017)

### 4.1 Improvement of depression-like behavior

MCI and depression are closely related, and depression and anxiety symptoms are considered to be important risk factors for the progression of MCI to dementia (Dean et al., 2014). About 35%–85% of patients with MCI have neuropsychiatric symptoms in addition to cognitive impairment (Gallagher et al., 2017). Neuropsychiatric symptoms include depression, apathy, anxiety, and irritability, with depression and anxiety symptoms as the most common. Patients with MCI are more likely to be depressed than those with normal cognitive function, with depressive symptoms occurring in 26.3% and 18.0%, respectively (Dooley et al., 2020; Hidaka et al., 2012). Patients with MCI combined with a depressive mood have severe behavioral symptoms and verbally provocative behavior (Van der Mussele et al., 2013). Therefore, the assessment of depressive and anxiety symptoms and early intervention in patients with MCI are of great importance to prevent the deterioration of MCI and prevent dementia (Wiesli et al., 2017). The antidepressant-like and anxiolytic-like effects of an ethanolic extract of *T. Chebula* fruit were studied, and test results revealed significant improvements in mice (Mani et al., 2021). Additionally, the acute anxiolytic activity of an aqueous extract of *T. Chebula* was evaluated; the findings showed that the extract reduced fatigue, increased exploratory behavior, and relieved anxiety, thereby performing comparably with the standard anxiolytic drug valium (Chandrashekar et al., 2013). Furthermore, the antidepressant activity and mechanistic effects of the aqueous extract of *T. Chebula* were investigated; the results demonstrated that higher doses of the extract exhibited antidepressant activity. This outcome is likely mediated through the monoaminergic pathway (Dattatray et al., 2014). *T. Chebula* contains ellagic acid **59**, a rich source of polyphenolic di-lactones with significant anti-inflammatory and neuroimmunomodulatory activities. The antidepressant effect of ellagic acid was evaluated using a chronic unpredictable mild stress model in mice. Ellagic acid improved neuroendocrine and inflammatory responses in the model mice and exerted a notable antidepressant effect. Additionally, ellagic acid can improve anxiety, possibly by inhibiting toll-like receptor 4 and activating NF-E2-related factor, to reduce anxiety (Huang, 2020; Wang et al., 2020b). The anxiolytic effects of *T. Chebula* tannin extract was investigated using a heroin-induced anxiety model in mice. The outcomes demonstrated that the tannin extract acts as a natural product for neurodegenerative illnesses and exhibits substantial anxiolytic activity (Chandrasekhar et al., 2018).

### 4.2 Improvement of memory and cognitive level

Memory impairment and cognitive deficits are the primary clinical manifestations of MCI, with memory impairment as the core problem (Peritogiannis et al., 2022). Patients with MCI and memory impairment have a 38% risk of developing Alzheimer’s disease (Irish et al., 2011). Currently, trials indicate that memory training, a feasible non-pharmacological intervention, can improve cognitive function in patients with MCI and result in positive performance changes in older adults who have cognitive impairment (Olchik et al., 2013). Corilagin **29**, extracted from *T. Chebula*, significantly improved sleep deprivation-induced memory impairment, which was associated with the inhibition of NADPH oxidase 2 and the activation of NF-E2-related factor (Wang et al., 2020a). Polyphenolic compounds, such as quercetin **129** and rutin **131**, demonstrated potential protective effects on inhibitory avoidance memory deficits in zebrafish, suggesting their use in preventing and treating neurodegenerative diseases (Richetti et al., 2011). Rutin prevents morphological changes in hippocampal tissue by lowering the expression of cyclooxygenase-2, glial fibrillary acidic protein, interleukin-8, and inducible nitric oxide synthase, thereby attenuating STZ-induced inflammatory responses, as demonstrated in a rat model of cognitive impairment (Javed et al., 2012). Studies have confirmed that rutin can prevent cognitive problems. A passive avoidance learning memory test assessed learning and memory in normal and diabetic rats after 30 days of rutin administration and revealed enhanced cognitive performance in both groups (Hasanein et al., 2020). Additionally, rutin exhibits multi-targeted therapeutic potential for cognitive deficits associated with chronic cerebral hypoperfusion (Qu et al., 2014). Insulin deficiency in the brain, or “diabetes in the brain”, potentially caused by a high-cholesterol diet, is associated with cognitive dysfunction and neurodegenerative diseases. Rutin may reverse high cholesterol-induced inflammatory changes, apoptosis activation, and cognitive deficits (Tomycz and Friedlander, 2011; Sun et al., 2021). Furthermore, ellagic acid **59** can hinder scopolamine and diazepam-induced cognitive impairment, indicating its potential as a memory restorer in dementia treatment and in improving cognitive behavior (Jha et al., 2018; Mansouri et al., 2016).

### 4.3 Antioxidant effect

*T. Chebula* and its chemical constituents can improve the pathogenesis of various neurodegenerative diseases. Tannins, a unique class of phytochemicals in *T. Chebula*, exhibit a broad range of potential health benefits, particularly due to their antioxidant capacity (Hussain et al., 2018a; Kennedy and Wightman, 2011). This antioxidant property is valuable because it reduces oxidative damage, a major marker of almost all diseases (Hussain et al., 2018b). *T. Chebula* is rich in plant polyphenols, with a total polyphenol content reaching 13.27%. These polyphenols contain phenolic hydroxyl groups that can be dehydrogenated and oxidized during lipid peroxidation in cell membranes. This reaction provides electrons to reactive oxygen species (ROS), thereby effectively terminating the chain reaction of free radicals and protecting cell structures from damages (Gupta et al., 2020). Polyphenols in *T. Chebula* can effectively scavenge -OH radicals and significantly inhibit lecithin lipid peroxidation damage, with antioxidant effects comparable with those of rutin **131**, which are known for its strong antioxidant properties. The tannin (polyphenolic) antioxidant content of Asian ellagic acid in *T. Chebula* is up to 32%, which is closely related to the high antioxidant properties of *T. Chebula* (Chang and Lin, 2012). In a model of sodium oxalate-induced renal oxidative imbalance in female rats, *T. Chebula* extract (TCE) significantly increased the catalase (CAT), total reduced glutathione (GSH), and superoxide dismutase (SOD) activity, while reducing lipid peroxidation (LPO) induced by sodium oxalate (Saha and Verma, 2015). Increased inflammatory mediators can impair cognitive function and predispose individuals to neurological disorders. Pro-inflammatory cytokines and ROS production lead to oxidative stress, making antioxidants a potential therapeutic approach for these disorders. Ellagic acid (EA) stands out among antioxidants; it modulates immune response by significantly reducing glial cell expression, attenuating phosphorylated Tau and oxidative damage, improving the antioxidant system, and preventing increased acetylcholinesterase activity (Dornelles et al., 2020). The antioxidant effects of EA are due to its free radical scavenging properties and enhancement of endogenous antioxidants, such as GSH, SOD, catalase, glutathione reductase, and glutathione peroxidase (Javed et al., 2021). Sleep disorders (SD) can cause neurobehavioral deterioration, including cognitive impairment and memory deficits. Pilot studies found that EA reduced damages by activating the Nrf2/HO-1 pathway and inhibiting the TLR4-induced inflammatory response. Nrf2 regulates the antioxidant system and can be activated in response to oxidative stress (Butterfield et al., 2023). EA exerts antioxidant effects by activating Nrf2, protecting against mitochondrial dysfunction in rats through upregulation of Nrf2/HO-1, and inhibition of NF-κB signaling pathways (Wang et al., 2020b). In summary, the active ingredients of *T. Chebula*, primarily tannins and phenolic acids, exert their antioxidant effects by scavenging free radicals, affecting oxidative enzyme activity, and combating oxidative stress.

### 4.4 Anti-inflammatory effect

*T. Chebula* contains phenolic acid components, such as gallic acid **16**, which has significant anti-inflammatory activity. Gallic acid reduces neuroinflammation by decreasing Aβ plaque-associated microgliosis and astrocytosis, decreasing cytokine levels in microglia, and protecting neurons from Aβ-induced neurotoxicity by inhibiting NF-κB acetyltransferase. Tannic acid, a hydrolysable glycosidic polyphenol polymer of gallic acid, also exhibits anti-inflammatory effects, primarily by reducing brain lipid peroxidation (MDA) and pro-inflammatory cytokines (IL-1β, IL-6, and TNF-α) to improve memory and cognitive impairment (Zhang et al., 2017; Caruso et al., 2022; Mirshekar et al., 2018). *T. Chebula* is also rich in flavonoids, which inhibit neuroinflammation by directly reacting with and removing neurotoxic substances and pro-inflammatory factors produced in the brain as a result of aging (Calderaro et al., 2022; Spencer, 2010). Corilagin **29**, a member of the tannin family, has significant anti-inflammatory effects. Its intrinsic anti-inflammatory mechanism involves significantly reducing the production of pro-inflammatory cytokines and mediating TNF-α, IL-1β, IL-6, NO (iNOS), and COX-2 nuclear translocation by blocking NF-κB at the protein and gene levels (Zhao et al., 2008). These findings were further verified, showing that corilagin reduces NO production and the mortality of microglia stimulated by lipopolysaccharide (LPS), scavenges diphenyl-1-propenylhydrazyl (DPPH) radicals by 96%, and decreases malondialdehyde (MDA) levels, thereby inhibiting inflammatory and oxidative processes (Gaire et al., 2013). Studies have also evaluated the anti-inflammatory properties of TCE on LPS-induced inflammation in microglia. TCE significantly reduced LPS-induced inflammation, increased the anti-inflammatory rate in mouse macrophages, and influenced inflammation and anti-inflammatory mediators (Rahimi et al., 2018). TCE has been proven effective in healing neuroinflammatory disorders, indicating its potential as an anti-inflammatory agent in treating central nervous system inflammatory diseases.

### 4.5 Repairing synaptic plasticity damage

Plastic synapses may be crucial and unique biological neural networks associated with learning and memory and maintain stable adaptive properties in response to external environmental changes (Brown and Piscopo, 2013; Yang et al., 2018). Increasing evidence indicates that phytochemicals have multiple biochemical functions (Skaper et al., 2017). For instance, quercetin **129**, found in *T. Chebula*, significantly reduces memory impairment in rats. Quercetin improves mitochondrial production, structure, and function in rat hippocampal neurons under low-pressure and low-oxygen conditions, significantly reducing synaptic structural damage. It also regulates the expression of fusion and fission-related proteins in mitochondria and promotes the expression of brain-derived neurotrophic factor (Liu, 2016). Quercetin can repair lead-induced synaptic plasticity damage in the hippocampus. It reduces synaptic plasticity damage in the DG region of rats exposed to chronic lead, suggesting that quercetin could be a potential treatment for lead-induced cognitive impairment (Hu, 2008). The colorimetric method of tetramethylazolium salt (MTT) and DAPI staining indicated that quercetin increased the survival rate of PC12 cells in the glutamate-treated group in a concentration- and time-dependent manner. Quercetin may protect against glutamate-induced neurological damage by inhibiting outward potassium currents in hippocampal pyramidal neurons, indicating its reparative effects on nerves (Zhao et al., 2010). Additionally, ellagic acid **59**, in *T. Chebula*, alleviates synaptic plasticity impairment in rats with acute kidney injury (AKI). Treatment with ellagic acid improved brain electrophysiology, spatial learning, and memory indices in AKI rats (Sarkaki et al., 2022).

### 4.6 Anti-apoptotic effect

Apoptosis of nerve cells significantly impacts neurodegenerative diseases. Flavonoids in *T. Chebula* exhibit various neuroprotective effects and improve cognitive dysfunction by interacting with key proteins and lipid kinase signaling cascades, thereby inhibiting apoptosis triggered by neurotoxic species and promoting neuronal survival (Vauzour et al., 2008). This cytoprotective ability is likely related to their anti-apoptotic properties. Studies using H_2_O_2_-treated PC12 cells created a model of apoptosis, characterized by cell death not accompanied by LDH release (Dajas et al., 2003). The ethanolic extract of *T. Chebula* fruit effectively inhibited COX-1, COX-2, and 5-LOX; mechanistic studies showed that *T. Chebula* acts as a dual inhibitor of COX-2 and 5-LOX and can inhibit apoptosis (Reddy et al., 2009). *T. Chebula* also contains ellagic acid **59**, which plays an anti-apoptotic role in neurodegenerative diseases. Pilot studies demonstrated that ellagic acid significantly improved spatial learning and memory deficits and reduced neuronal apoptosis and Aβ deposition in the hippocampus (Zhong et al., 2018). Ellagic acid also inhibits tau hyperphosphorylation and reduces glycogen synthase activity, and these effects are partially mediated by the AKT/GSK3β signaling pathway. Rutin **131**, another flavonoid in *T. Chebula*, was investigated for its effect on isoflurane-induced apoptosis. It significantly decreased isoflurane-induced apoptosis, as measured by TUNEL assays, and increased the expression levels of caspase-3, Bad, Bax, and MAPK proteins. Rutin provided neuroprotection against isoflurane-induced neuronal apoptosis and improved learning and memory in rats by effectively regulating MAPK protein expression levels (Li et al., 2017).

## 5 Safety evaluation

*T. Chebula* is widely used in conventional and modern medicine, and related safety studies are crucial for determining safe doses for further clinical trials. Various studies have evaluated the *in vitro* and *in vivo* toxicity of methanolic extracts of *T. Chebula* fruit (TCF) and skin (TAB) using cytotoxicity, hemolytic activity, mutagenicity, and genotoxicity tests (Suganthy et al., 2018). Acute and subacute toxicity studies indicated that oral administration of TCF and TAB is relatively non-toxic. Specifically, aqueous, ethanol, and ethyl acetate extracts of TCF showed no cytotoxicity to sheep erythrocytes. Acute oral toxicity tests in rats demonstrated that aqueous extracts from dried TCF had no acute or chronic toxicity in either female or male rats (Sireeratawong et al., 2013). Additionally, the mutagenicity of the *T. Chebula* ethyl acetate (EtOAc) soluble fraction was assessed *in vitro*, and a continuous 14-day oral administration experiment revealed no adverse effects on rats at a dose of 2000 mg/kg (Kim et al., 2012). Furthermore, *T. Chebula* had genotoxic effects in the VITOTOX test and the Ames test (Bag et al., 2013).

## 6 Quality control

The quality control level of *T. Chebula* needs improvement. The 2020 edition of the Chinese Pharmacopoeia lacks requirements for content determination, leading to gaps in quality control for *T. Chebula* herbs. In this regard, a high-performance liquid chromatography (HPLC) method was established to determine chebulagic acid and chebulinic acid in *T. Chebula*; it enhanced the content determination guidelines in the 2020 Chinese Pharmacopoeia and facilitated improved quality control of *T. Chebula* herbs (Wang et al., 2024). Quality testing standards typically focus on a few key components in *T. Chebula*. The total tannin content of *T. Chebula* was determined using HPLC-DAD analysis, and active site profiles were constructed. This method allows for accurate identification and quality control of various types of *T. Chebula* available in the market (He et al., 2017). Near-infrared (NIR) diffuse reflectance spectroscopy, which is widely used for rapid detection, was combined with partial least square regression (PLS) to establish a quantitative model for gallic acid determination in *T. Chebula*. The results showed no significant difference between the predicted and conventional methods, indicating the feasibility of this technique for *T. Chebula* quality determination (Luo et al., 2016). Additionally, an HPLC method was developed for the simultaneous determination of several key constituents in chebulanic herbs: gallic acid **16**, punicalagin **39**, punicalagin B **77**, methyl gallate **17**, corilagin **29**, pentagalloylglucose **82**, and ellagic acid **59**. The concentrations were 0.186–3.720 μg for gallic acid, 0.046–0.552 μg for punicalagin A, 0.029–0.192 μg for punicalagin B, 0.182–2.730 μg for methyl gallate, 0.069–1.388 μg for corilagin, 0.324–6.480 μg for pentagalloylglucose, and 0.167–1.672 μg for ellagic acid, and all of which showed good linearity (Qi, 2018).

## 7 Conclusion and future perspectives

MCI poses significant hazards, including cognitive decline, psychological health issues, decreased ability to perform daily activities, reduced social engagement, risk of progression to dementia, and burden on families (De Leon et al., 1997). MCI is a high-risk stage for Alzheimer’s disease, with some patients with MCI potentially progressing to dementia within a few years, leading to severe cognitive and functional impairments (Forrester et al., 2016; Kohler et al., 2016). Tibetan medicine offers advantages in treating MCI, such as holistic regulation, individualized treatment, high safety, symptom improvement, and slowing disease progression. By comprehensively applying traditional Tibetan therapeutic methods, effective interventions can be provided to patients with MCI to improve their quality of life and delay disease progression. MCI falls under the category of “Jie Xie Syndrome” in Tibetan medicine, which believes that regulating the imbalance of “Long” can treat “Jie Xie Syndrome.”

This article primarily reviews the active components and pharmacological effects of *T. Chebula* in treating MCI. We identified 171 compounds from *T. Chebula*, including 83 tannins, 16 phenolic acids, 6 flavonoids, 29 triterpenoids, 17 volatile oils, and 20 other compounds. Among these, 11 compounds significantly improve MCI symptoms. The pharmacological activities of *T. Chebula* that help alleviate MCI symptoms mainly include antioxidant, hypoglycemic, antiviral, and anti-inflammatory effects. However, current research on the quality standards of *T. Chebula* is insufficient, and the specific chemical components of *T. Chebula* need further exploration. Scholars need to conduct advanced research into the extraction and isolation of the chemical constituents of *T. Chebula*.

Neurodegenerative diseases involve oxidative stress, inflammation, abnormal protein accumulation, and mitochondrial dysfunction, thereby disrupting physiological processes and contributing to various neurological disorders, including depression. *T. Chebula* shows promise in alleviating depression and anxiety symptoms in MCI. Its active components can potentially reverse inflammation and apoptosis induced by high cholesterol and improve cognitive function and memory in patients with MCI. The neuroprotective effects of *T. Chebula* originate from its potent anti-inflammatory and antioxidant properties. It shields neurons from oxidative stress and neurotoxicity, reduces neuronal inflammation, and supports synaptic plasticity while enhancing cerebral blood flow. Moreover, *T. Chebula* appears beneficial for sleep, a critical issue in MCI, although additional research is needed to fully understand its mechanisms in improving sleep quality. In summary, *T. Chebula* exhibits diverse pharmacological effects including antioxidant, anti-inflammatory, synaptic plasticity repair, anti-apoptotic, and enhancement of depression-like behavior, memory, and cognitive function. Toxicological studies, encompassing cytotoxicity, hemolytic activity, mutagenicity, and genotoxicity tests, indicate the relative safety of *T. Chebula* in *in vitro* and *in vivo* settings. However, further refinement of the specific mechanisms underlying these pharmacological actions is necessary, as well as research into optimal dosage control. Experimental studies using various animal models and cell lines support the potential of *T. Chebula* in alleviating symptoms of MCI and achieving specific therapeutic goals. However, the majority of these studies are confined to cellular and animal research, with limited clinical trials conducted. This gap impedes the validation of the efficacy of *T. Chebula* in humans and poses challenges for the development of new treatments based on *T. Chebula*.

Despite the progress in the study of MCI, treating MCI still faces multiple challenges and issues. First, MCI diagnostic results are inconsistent due to the lack of uniform early diagnostic criteria and the difficulty of screening (Roberts and Knopman, 2013). Its mild symptoms are not easily recognized, which may cause patients to miss the optimal intervention period. Second, the effectiveness and application of treatment methods are limited. Although various methods, such as drug therapy, cognitive training, and lifestyle interventions, exist, they have its limitations (Eshkoor et al., 2015). Drug treatments, such as the use of cholinesterase inhibitors and NMDA receptor antagonists, may show some effects but generally have limited efficacy and may come with side effects. The development of multi-component, multi-target traditional Tibetan medicines with therapeutic advantages, such as Tibetan medicine *T. Chebula*, is lacking. Non-drug treatments, such as cognitive training, exercise, and dietary adjustments, have potential but their effects vary from person to person and lack large-scale, long-term validation. Third, the lack of individualized treatment is a significant issue. The causes, symptoms, and progression rates of patients with MCI vary, but current treatments often use a uniform approach and lack tailored treatments for individual differences. Fourth, treatment adherence and long-term management issues deserve attention. Patients with MCI may lack the motivation and adherence to persist with long-term treatments and interventions, thereby affecting the treatment outcomes (Portet et al., 2006). Long-term management is challenging because MCI is a gradual, progressive condition that requires continuous monitoring and management. Fifth, the inadequacy of psychological and social support systems affects overall treatment outcomes. Patients with MCI and their families often face psychological stress and anxiety, but psychological support and counseling services are relatively insufficient. Additionally, social support systems and resources for patients with MCI are limited, thereby affecting their quality of life and social participation (Jongsiriyanyong and Limpawattana, 2018). Finally, research and development of related drugs face numerous challenges. A deeper understanding of the pathogenesis of MCI is still lacking, which limits the development of new treatments. MCI involves multiple pathological mechanisms, including neuroinflammation, oxidative stress, and metabolic abnormalities. Single-action mechanism drugs often struggle to effectively treat these complexities. Furthermore, clinical trials require long-term follow-up and large sample sizes due to the mild and slow-progressing nature of MCI symptoms, thereby increasing the difficulty and cost of research (Hendrix, 2012). In summary, treating MCI involves numerous issues, such as difficulty in early diagnosis, limited treatment effectiveness, lack of individualized treatment, challenges in adherence and long-term management, inadequate psychological and social support, and research and development challenges.

To address the numerous challenges in treating MCI, future research should focus on multiple aspects to advance therapeutic methods. First, developing and promoting standardized diagnostic tools is essential to ensure diagnostic accuracy and consistency. Initiatives include increasing public and medical personnel awareness of early MCI symptoms and the importance of early detection through regular screening. Second, efforts should be intensified in developing new drugs, especially those targeting the pathological mechanisms of MCI. Exploring the combination of pharmacological and non-pharmacological interventions, such as cognitive training, exercise, and dietary adjustments, to form comprehensive treatment plans can enhance efficacy (Poptsi et al., 2022). Additionally, research and development should be strengthened for traditional medicines with multi-component and multi-target actions, such as Tibetan medicine *T. Chebula* (Klimova et al., 2019). Third, the importance of personalized treatment must be emphasized. Treatment plans should be tailored to the specific conditions of each patient, including the choice of medication and lifestyle interventions. Research on biomarkers should be strengthened to develop predictive tools that help identify high-risk patients and guide personalized treatment. Fourth, psychological counseling and support services should be enhanced to provide necessary emotional support and counseling for patients with MCI and their families. Establishing and improving social support networks can offer various forms of assistance and resources to patients with MCI, thereby improving their quality of life and social participation. Finally, promoting interdisciplinary collaboration is crucial for in-depth research into the pathological mechanisms of MCI. Establishing large-scale MCI databases that integrate genomic, proteomic, and metabolomic data can help uncover potential pathological mechanisms and biomarkers. Utilizing artificial intelligence and machine learning techniques to analyze complex data can reveal new research directions and therapeutic targets. Innovative clinical trial designs, such as adaptive and crossover designs, should be employed to increase trial efficiency and provide effective treatment options for MCI. In summary, addressing the challenges of MCI treatment requires a multifaceted approach, including the development of standardized diagnostic tools, new drug development, personalized treatment plans, enhanced psychological and social support, and interdisciplinary research to uncover new therapeutic targets and improve treatment efficacy.

As future research progresses, Tibetan medicine *T. Chebula* shows immense promise in treating MCI and offers potential benefits to patients by improving the overall human quality of life.

## Glossary

*T. Chebula*: *Terminalia chebula* Retz.
TCM: traditional Chinese medicine
MCI: mild cognitive impairment
TCE: *T. Chebula* extract
TCF: *T. Chebula* fruit
EtOAc: ethyl acetate
NPS: neuropsychiatric symptoms
MAO-A: monoamine oxidase alpha
FST: forced swim test
TST: tail suspension test
EA: Ellagic acid
CUMS: chronic unpredictable mild stress model
EPM: elevated plus maze
OFT: open field test
LDT: light/dark box test
VCT: Vogel’s conflict test
MT: memory training
COX-2: cyclooxygenase-2
GFAP: glial fibrillary acidic protein
IL-8: interleukin-8
iNOS: inducible nitric oxide synthase
SOD: superoxide dismutase
LPO: lipid peroxide
ROS: reactive oxygen species
BDNF: brain-derived neurotrophic factor
MCAO: middle cerebral artery occlusion
NO: nitric oxide
PC12: pheochromocytoma cell
SD: sleep disorders
DPPH: diphenyl-1-propenylhydrazyl
LPS: lipopolysaccharide
